# Supplementary material for: SCOPE: Revealing Hidden Mechanisms in Phenotypic Screens Through Target and Pathway Enrichment
Source: bioRxiv. 2025 Jul 14:2025.07.11.664427. Preprint. [Version 1] doi: 10.1101/2025.07.11.664427 (PMC12338551; doi:10.1101/2025.07.11.664427)
Supplement: Supplement 1 [file media-1.pdf]

Supplementary Table 1. List of enriched targets after target enrichment analysis. All targets with a 5% cutoff for FDR and p-value are listed.

Uniprot ID	Symbol	Protein Name	Fisher_p-value	FDR (BH)
P28223	HTR2A	5-hydroxytryptamine receptor 2A	1.58E-10	6.20E-08
P28335	HTR2C	5-hydroxytryptamine receptor 2C	2.25E-07	3.68E-05
P08912	CHRM5	Muscarinic acetylcholine receptor M5	2.82E-07	3.68E-05
P35348	ADRA1A	Alpha-1A adrenergic receptor	4.86E-07	4.76E-05
P08172	CHRM2	Muscarinic acetylcholine receptor M2	8.27E-07	6.48E-05
P35462	DRD3	D(3) dopamine receptor	1.30E-06	8.52E-05
P21728	DRD1	D(1A) dopamine receptor	3.30E-06	1.85E-04
P20309	CHRM3	Muscarinic acetylcholine receptor M3	4.59E-06	2.25E-04
P50406	HTR6	5-hydroxytryptamine receptor 6	6.91E-06	3.01E-04
Q12809	KCNH2	Potassium voltage-gated channel subfamily H member 2	7.73E-06	3.03E-04
P08173	CHRM4	Muscarinic acetylcholine receptor M4	2.29E-05	8.18E-04
P14416	DRD2	D(2) dopamine receptor	6.35E-05	1.92E-03
P25100	ADRA1D	Alpha-1D adrenergic receptor	6.17E-05	1.92E-03
P41595	HTR2B	5-hydroxytryptamine receptor 2B	7.63E-05	2.14E-03
P08908	HTR1A	5-hydroxytryptamine receptor 1A	1.45E-04	3.54E-03
P11229	CHRM1	Muscarinic acetylcholine receptor M1	1.37E-04	3.54E-03
P23975	SLC6A2	Sodium-dependent noradrenaline transporter	1.79E-04	3.89E-03
P08913	ADRA2A	Alpha-2A adrenergic receptor	1.78E-04	3.89E-03
P18089	ADRA2B	Alpha-2B adrenergic receptor	2.19E-04	4.52E-03
P25021	HRH2	Histamine H2 receptor	2.62E-04	4.89E-03
P35372	OPRM1	Mu-type opioid receptor	2.61E-04	4.89E-03
P35367	HRH1	Histamine H1 receptor	3.23E-04	5.51E-03
P31645	SLC6A4	Sodium-dependent serotonin transporter	3.11E-04	5.51E-03
P46098	HTR3A	5-hydroxytryptamine receptor 3A	7.44E-04	1.22E-02
P21918	DRD5	D(1B) dopamine receptor	1.43E-03	2.25E-02
P41145	OPRK1	Kappa-type opioid receptor	3.12E-03	4.71E-02
P18825	ADRA2C	Alpha-2C adrenergic receptor	3.65E-03	5.29E-02

Uniprot ID	Symbol	Protein Name	Fisher_p-value	FDR (BH)
P21917	DRD4	D(4) dopamine receptor	4.42E-03	6.18E-02
Q99720	SIGMAR1	Sigma non-opioid intracellular receptor 1	4.70E-03	6.35E-02
Q9NY46	SCN3A	Sodium channel protein type 3 subunit alpha	5.72E-03	7.47E-02
P08183	ABCB1	Multidrug resistance protein 1	6.88E-03	8.70E-02
Q99250	SCN2A	Sodium channel protein type 2 subunit alpha	8.72E-03	9.76E-02
P35498	SCN1A	Sodium channel protein type 1 subunit alpha	8.04E-03	9.76E-02
Q14147	DHX34	Probable ATP-dependent RNA helicase DHX34	8.70E-03	9.76E-02
Q14562	DHX8	ATP-dependent RNA helicase DHX8	8.70E-03	9.76E-02
P47898	HTR5A	5-hydroxytryptamine receptor 5A	1.29E-02	1.41E-01
P04156	PRNP	Major prion protein	1.68E-02	1.73E-01
Q7Z2K8	GPRIN1	G protein-regulated inducer of neurite outgrowth 1	1.68E-02	1.73E-01
P34969	HTR7	5-hydroxytryptamine receptor 7	2.15E-02	1.74E-01
P25103	TACR1	Substance-P receptor	2.34E-02	1.74E-01
Q08499	PDE4D	cAMP-specific 3',5'-cyclic phosphodiesterase 4D	2.10E-02	1.74E-01
Q16769	QPCT	Glutaminyl-peptide cyclotransferase	2.97E-02	1.74E-01
Q86TP1	PRUNE1	Exopolyphosphatase PRUNE1	2.97E-02	1.74E-01
P20648	ATP4A	Potassium-transporting ATPase alpha chain 1	2.20E-02	1.74E-01
Q05586	GRIN1	Glutamate receptor ionotropic, NMDA 1	1.96E-02	1.74E-01
P28907	CD38	ADP-ribosyl cyclase/cyclic ADP-ribose hydrolase 1	2.97E-02	1.74E-01
Q9Y5S1	TRPV2	Transient receptor potential cation channel subfamily V member 2	2.97E-02	1.74E-01
P51878	CASP5	Caspase-5	2.97E-02	1.74E-01
P49662	CASP4	Caspase-4	2.97E-02	1.74E-01
P62937	PPIA	Peptidyl-prolyl cis-trans isomerase A	2.97E-02	1.74E-01
Q08752	PPID	Peptidyl-prolyl cis-trans isomerase D	2.97E-02	1.74E-01
P23284	PPIB	Peptidyl-prolyl cis-trans isomerase B	2.97E-02	1.74E-01
P20815	CYP3A5	Cytochrome P450 3A5	2.04E-02	1.74E-01

Uniprot ID	Symbol	Protein Name	Fisher_p-value	FDR (BH)
O14939	PLD2	Phospholipase D2	2.97E-02	1.74E-01
Q13393	PLD1	Phospholipase D1	2.97E-02	1.74E-01
P10909	CLU	Clusterin	2.97E-02	1.74E-01
Q96LB1	MRGPRX2	Mas-related G-protein coupled receptor member X2	2.97E-02	1.74E-01
Q13639	HTR4	5-hydroxytryptamine receptor 4	2.65E-02	1.74E-01
P49069	CAMLG	Calcium signal-modulating cyclophilin ligand	2.97E-02	1.74E-01
Q96LZ3	PPP3R2	Calcineurin subunit B type 2	2.97E-02	1.74E-01
P30405	PPIF	Peptidyl-prolyl cis-trans isomerase F, mitochondrial	2.97E-02	1.74E-01
Q9H4I9	SMDT1	Essential MCU regulator, mitochondrial	2.97E-02	1.74E-01
Q9HAW9	UGT1A8	UDP-glucuronosyltransferase 1-8	2.20E-02	1.74E-01
Q6ICB4	PHETA2	Sesquipedalian-2	2.97E-02	1.74E-01
Q2M1K9	ZNF423	Zinc finger protein 423	2.97E-02	1.74E-01
Q9NZI2	KCNIP1	Kv channel-interacting protein 1	2.97E-02	1.74E-01
Q01726	MC1R	Melanocyte-stimulating hormone receptor	2.97E-02	1.74E-01
Q92847	GHSR	Growth hormone secretagogue receptor type 1	4.10E-02	2.36E-01
P07550	ADRB2	Beta-2 adrenergic receptor	4.74E-02	2.68E-01
Q15125	EBP	3-beta-hydroxysteroid-Delta(8),Delta(7)-isomerase	4.78E-02	2.68E-01

Supplementary Table 2. Biological activity of bromocriptine against different human receptors.

Data taken from IUPHAR (https://www.guidetopharmacology.org/GRAC/LigandDisplayForward?tab=biology&ligandId=35).

**Target**	**Action**	**Value**	**Parameter**
ADRA2A	Antagonist	8.0 – 8.3	pK_i
HTR1D	Partial agonist	8	pK_i
HTR1A	Partial agonist	7.9	pK_i
DRD2	Full agonist	7.3 – 8.3	pK_i
DRD3	Partial agonist	7.1 – 8.2	pK_i
ADRA2C	Antagonist	7.6	pK_i
HTR6	Full agonist	7.5	pK_i
HTR2B	Antagonist	7.3	pK_i
ADRA2B	Antagonist	6.9 – 7.5	pK_i
HTR2A	Partial agonist	7	pK_i
HTR1B	Partial agonist	6.5	pK_i
DRD4	Antagonist	6.4	pK_i
DRD5	Full agonist	6.3	pK_i
DRD1	Partial agonist	6.2	pK_i
HTR2C	Partial agonist	6.1	pK_i
